# The S-Layer Glycoprotein of the Crenarchaeote *Sulfolobus acidocaldarius* Is Glycosylated at Multiple Sites with Chitobiose-Linked *N*-Glycans

**DOI:** 10.1155/2010/754101

**Published:** 2010-09-29

**Authors:** Elham Peyfoon, Benjamin Meyer, Paul G. Hitchen, Maria Panico, Howard R. Morris, Stuart M. Haslam, Sonja-Verena Albers, Anne Dell

**Affiliations:** ^1^Division of Molecular Biosciences, Faculty of Natural Sciences, Imperial College London, London SW7 2AZ, UK; ^2^Molecular Biology of Archaea, Max Planck Institute for Terrestrial Microbiology, Karl-von-Frisch-Straße 10, 35043 Marburg, Germany; ^3^Centre for Integrative Systems Biology, Faculty of Natural Sciences, Imperial College London, London SW7 2AZ, UK

## Abstract

Glycosylation of the S-layer of the crenarchaea *Sulfolobus acidocaldarius* has been investigated using glycoproteomic methodologies. The mature protein is predicted to contain 31 N-glycosylation consensus sites with approximately one third being found in the C-terminal domain spanning residues L_1004_-Q_1395_. Since this domain is rich in Lys and Arg and therefore relatively tractable to glycoproteomic analysis, this study has focused on mapping its N-glycosylation. Our analysis identified nine of the 11 consensus sequence sites, and all were found to be glycosylated. This constitutes a remarkably high glycosylation density in the C-terminal domain averaging one site for each stretch of 30–40 residues. Each of the glycosylation sites observed was shown to be modified with a heterogeneous family of glycans, with the largest having a composition Glc_1_Man_2_GlcNAc_2_ plus 6-sulfoquinovose (QuiS), consistent with the tribranched hexasaccharide previously reported in the cytochrome b_558/566_ of *S. acidocaldarius*. *S. acidocaldarius* is the only archaeal species whose N-glycans are known to be linked via the chitobiose core disaccharide that characterises the N-linked glycans of *Eukarya*.

## 1. Introduction

In many *Archaea *the surface layer (S-layer) proteins are the sole cell wall component [1]. These S-layer proteins assemble into a natural 2-D crystal structure with very strong self interactions. In *Archaea*, which do not possess other cell wall components, the S-layer has to maintain the cell integrity and stabilize as well as to protect the cell against mechanical and osmotic stresses or extreme pH conditions. It is also predicted that the S-layer has to maintain or even determine the cell shape [2–6].

In *Sulfolobus* spp. the S-layer is composed of two proteins: a small protein of approximately 45 kD, SlaB, and a large protein, SlaA, of approximately 120 kDa. SlaB is an integral membrane protein and its strong interaction with SlaA, which covers the whole cell surface, tethers the S-layer to the membrane [8, 9]. Taking into account the harsh growth condition of the thermoacidophilic *Sulfolobus* spp. (pH 2-3 and 75–80°C), the S-layer proteins will play an important role in maintaining cell integrity and must be adapted to be functional under these conditions. 

One possible posttranslational modification proteins can undergo is glycosylation, which has a major effect on stability and half-life [10]. Indeed, all archaeal S-layer proteins which have been structurally studied to date, have been found to carry N-glycans [11–17].

Although *Eukarya*, *Bacteria*, and *Archaea* all share certain characteristics of the N-glycosylation pathway, the resulting glycan structures in *Bacteria* and *Archaea *are more diverse than in *Eukarya * [18, 19]. Notably a far greater variety of monosaccharides is used, many of which carry functionalities such as sulfate and methyl groups, or even amino acids such as threonine [12]. In the last two years, substantial progress in describing the enzymes involved in archaeal N-glycosylation pathways has been made [19–23]. The archaeal N-glycosylation machinery combines aspects of both the eukaryal and bacterial pathways. For instance *Archaea *and *Eukarya* use dolichol as the lipid carrier whereas *Bacteria* use undecaprenyl. On the other hand, in *Archaea* and *Bacteria* the oligosaccharyltransferase is comprised of a single subunit whereas in *Eukarya* it is a multimeric complex.

So far proteins from about 25 species of *Archaea* have been reported to be glycosylated and about ten have had their N-glycans partially or fully characterized [19, 24]. The best understood are S-layers of the halophiles *Halobacterium salinarum* and *Haloferax volcanii*, and S-layers and flagellins of the methanogens *Methanothermus fervidus*, *Methanococcus voltae,* and *M. maripaludis*. In contrast to the N-glycans of *Eukarya,* which are almost always branched and usually considerably greater than six residues in size, these archaeal glycoproteins contain unbranched glycans most of which have fewer than six sugar residues. The exception is *Hbt. salinarum* which, in addition to bearing a trisaccharide composed of monosulfated glucuronic acid linked via glucose at about ten consensus sites, has one N-linked site occupied by a GalNAc-linked polysaccharide comprised of multiple repeats of a sulfated pentasaccharide (composed of GlcNAc, GalNAc, Gal, GalA, and 3-O-methyl-GalA) [25]. In *H. volcanii* the S-layer glycoprotein is modified by the attachment of a pentasaccharide, composed of two hexoses, two hexuronic acids, and a methylester of hexuronic acid [26, 27]. The N-glycans attached to the S-layer of *Methanothermus fervidus* are hexasaccharides containing 3-O-methylmannose, mannose, and GalNAc [28]. In* M. voltae *the flagellins and S-layer proteins are glycosylated with a complex trisaccharide composed of GlcNAc, GlcdiNAcA, and a threonine-substituted ManNAcA [12]. A second strain of *M. voltae* has been recently found to carry tetrasaccharides which share this trisaccharide sequence capped by an uncharacterized sugar [29]. An even more unusual tetrasaccharide has been found on the pilins and flagellins of* M. maripaludis*. This has the sequence Sug-4-*β*-ManNAc3NAmA6Thr-4-*β*-GlcNAc3NAcA-3-*β*-GalNAc where Sug is a highly unusual aldolase diglycoside [30].

Glycosylation of extreme thermophile members of the *Archaea* domain is quite poorly understood despite the fact that one member of this class, *Thermoplasma acidophilum*, from the Euryarchaeota kingdom of *Archaea*, was amongst the first of the archaea to have its glycoproteins studied by biophysical methods [31]. This early study, which reported the presence of branched mannose-rich glycans linked via GlcNAc to Asn, has not, however, been followed up with more rigorous structure analysis. A second extremophile member of the Euryarchaeota kingdom to have its glycosylation studied is *Pyrococcus furiosus*, which, interestingly, has also been shown to biosynthesis branched glycans [32]. The oligosaccharyltransferase from this species has been purified, and its ability to glycosylate a fluorescently labeled peptide containing a consensus sequence has been assayed in the presence and absence of lipid-linked oligosaccharide (LLO) prepared from *Pyrococcus furiosus* cells. In the presence of the LLO a glycopeptide was produced which was shown by mass spectrometry to be a branched heptasaccharide having a pentose sugar attached to each of the second and third residues of a pentasaccharide of sequence HexNAc-HexA-Hex-Hex-HexNAc [32].

**Figure 1:**
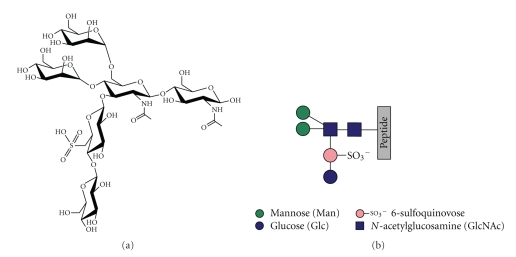
(a) Structure of the 6-sulfoquinovose-containing tribranched hexasaccharide identified in cytochrome b_558/566_ [7]. (b) This shows a symbolic representation of the glycan which is used in the annotations of Figures 4–6.

Branching is also a feature of the only glycan so far determined from a member of the Crenarchaeota kingdom. Thus cytochrome b_558/566_ of *Sulfolobus acidocaldarius*, which grows optimally at 75–80°C and pH 2-3, was shown to be modified with a tribranched hexasaccharide of composition Glc_1_Man_2_GlcNAc_2_ plus 6-sulfoquinovose, an unusual sugar which is characteristic of chloroplasts and photosynthetic bacteria (see Figure 1 for structure) [7]). As, to date, this is the only characterised glycan structure from a crenarchaeal species our objective is to determine the glycan composition of other extracellular proteins of the *Sulfolobales*. It is known that nearly all extracellular proteins found in these organisms are glycosylated [8, 33, 34]. As a first model protein, the S-layer protein of *S. acidocaldarius* was isolated and its glycosylation investigated using glycoproteomic methodologies.

## 2. Materials and Methods

### 2.1. Strains and Growth Conditions

*S. acidocaldarius * (DSM639) was grown in Brock medium at pH 3 and 76°C [35] and the medium was supplemented with 0.1(w/v) % of tryptone as sole carbon and energy source. Growth of cells was monitored by measuring the optical density at 600 nm.

### 2.2. S-Layer Isolation

Fresh cells or frozen cell pellets from a 50 ml culture were resuspended in 40 ml buffer A (10 mM NaCl, 1 mM PMSF, 0.5% Na-Lauroylsarcosine) with the addition of a little bit of DNAse. The samples were shaken for 45 minutes at 37°C and centrifuged for 20 min in an Optima Max-XU Ultracentrifuge (Beckman Coulter) at 16.000 rcf, yielding a brownish tan pellet. The pellet was resuspended in 1,5 ml buffer A and incubated for 30 min at 37°C. After centrifugation in a tabletop centrifuge at 14.000 rpm the pellet was purified by repeatable washes in buffer B (10 mM NaCl, 0,5 mM MgSO_4_, 0.5% SDS), incubation for 20 min at 37°C and subsequent centrifugation, until a translucent tan pellet was obtained. Once the pellet was translucent the S-layer proteins were once washed with water ad then stored in water at 4°C.

### 2.3. Proteolytic Digestion for Glycoproteomic Analysis

Purified S-layer samples of *S. acidocaldarius* were run on a 2–8% precast gel (Invitrogen, Paisley, UK) and stained with Novex Colloidal blue stain (Invitrogen). The S-layer was observed as a broadband between 116 and 160 kDa. The band was then excised and cut into pieces, destained using 400 *μ*l of 50% (v/v) acetonitrile in 0.1 M ammonium bicarbonate (pH 8.4) and dried in a SpeedVac. Reduction/carboxymethylation was carried out by swelling and incubating the dried gel pieced in Dithiothreitol (10 mM) (200 *μ*l) in ammonium bicarbonate (AMBIC) (50 mM, pH 8.4) (Roche, West Sussex, UK) at 56°C for 30 min. The DTT solution was then removed and the gel pieces were washed with acetonitrile (200 *μ*l) and dried. The gel pieces were incubated in dark at room temperature in 50 mM iodoacetic acid (IAA) (200 *μ*l) (Sigma-Aldrich Dorset, UK) which was dissolved in ammonium bicarbonate (50 mM, pH 8.4). The IAA was then removed and gel pieces were washed in AMBIC buffer (500 *μ*l) for 15 min. The AMBIC was removed and gel pieces were shrunk in acetonitrile (200 *μ*l) for 5 min. The gel pieces were dried in SpeedVac and then subjected to digestion with trypsin or chymotrypsin. For the tryptic digest the dried gel pieces were reswelled in AMBIC solution and incubated at 37°C with 25ng/*μ*l trypsin (20 *μ*l) (Promega cat V5111) overnight. The supernatant was removed and placed in a clean eppendorf. The gel pieces were then incubated in 0.1% TFA (50 *μ*l) at 37°C for 10 min. Acetonitrile (100 *μ*l) was added to the mixture which was incubated at 37°C for 15 min. The supernatant was then pooled with the previous supernatant and the process was repeated twice. The supernatant volume was then reduced (to about 35 *μ*l) in preparation for LC-MS. For the chymotryptic digest re-swelled gel pieces were incubated at 37°C in 25 ng/*μ*l chymotrypsin (Sigma C-3142) dissolved in Tris-HCl (100 mM, pH 7.8), overnight. The remainder of the experiment was carried out as for tryptic digestion.

### 2.4. LC-MS Analysis

The extracted peptides/glycopeptides from the gel pieces were analyzed a nano*-*LC-ES-MS/MS employing a quadrupole TOF mass spectrometer (Q-STAR Pulsar I, MDS Sciex). Separation of the peptides/glycopeptides was carried out by using a nano*-*LC gradient method generated by an Ultimate pump fitted with a Famos autosampler and a Switchos microcolumn switching module (LC Packings, Amsterdam, The Netherlands). The system was coupled to an analytical C_18_ nanocapillary (75 m inside diameter × 15 cm, PepMap) and a microprecolumn C_18_ cartridge for online peptide/glycopeptide separation. The digest was first loaded onto the precolumn and eluted with 0.1% formic acid (Sigma) in water (HPLC grade, Purite) for 4 min. The eluant was then transferred onto the column and eluted at a flow rate of 150 nL/min using the following gradient of solvent A [0.05% (v/v) formic acid in a 95:5 (v/v) water/acetonitrile mixture] and solvent B [0.04% formic acid in a 95:5 (v/v) acetonitrile/water mixture]: 99% A from 0 to 5 min, 99 to 90% A from 5 to 10 min, 90 to 60% A from 10 to 70 min, 60 to 50% A from 70 to 71 min, 50 to 5% A from 71 to 75 min, 5% A from 75 to 85 min, 5 to 95% A from 85 to 86 min, and 95% A from 86 to 90 min. Data acquisition was performed using Analyst QS software with an automatic information*-*dependent-acquisition (IDA) function.

### 2.5. Sugar Composition Analysis

Samples were hydrolysed in 1 M methanolic hydrogen chloride at 80°C for 16 h and the reagent was removed under a stream of nitrogen. Hexosamines were re-*N*-acetylated in 500 *μ*l of methanol/pyridine/acetic anhydride (500:1:5, v/v/v) for 15 min at room temperature, then dried under nitrogen. Trimethylsilyl derivatisation was performed in 200 *μ*L of Tri-Sil “Z” (Pierce) at room temperature for 30 min, after which the reagent was removed under nitrogen. Derivatized monosaccharides were resuspended in 1 ml of hexanes, centrifuged at 3000 rpm for 10 min, and the supernatant transferred and dried under nitrogen for analysis by gas chromatography-mass spectrometry (GC-MS).

### 2.6. GC-MS Analysis

This was carried out using a Perkin Elmer Clarus 500 instrument, fitted with a RTX-5 (30 m × 0.25 mm internal diameter, Restek Corp.). Temperature program: the oven was held at 65°C for 1 min before being increased to 140°C at a rate of 25°C/min, then to 200°C at a rate of 5°C/min and finally to a temperature of 300°C at a rate of 10°C/min.

## 3. Results

### 3.1. Strategy for Glycoproteomic Analysis of the *S. acidocaldarius* S-Layer

**Figure 2:**
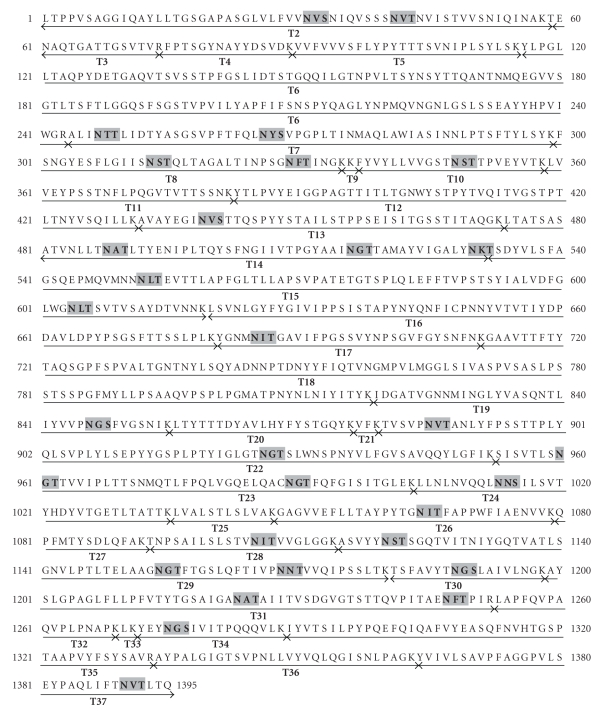
Polypeptide sequence of the *S. acidocaldarius* S-layer. The N-terminal signal sequence has been omitted. Consensus sequences for N-glycosylation are shaded and the predicted products of tryptic digestion are shown by underlining.

**Figure 3:**
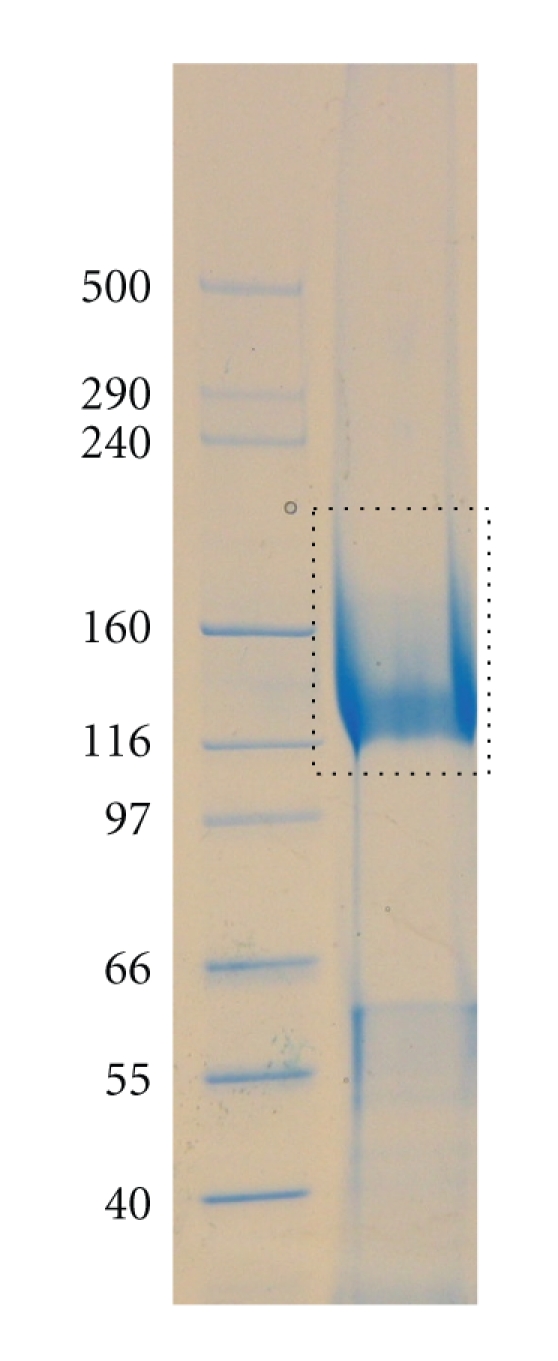
SDS PAGE gel of the *S. acidocaldarius* S-layer showing the band that was cut out for in-gel digestion.

The polypeptide sequence of the *S. acidocaldarius* S-layer SlaA protein (Saci_2355) is shown in Figure 2 [36]. The mature protein is predicted to comprise 1,395 amino acids and to contain 31 consensus sites for N-glycosylation. These are scattered throughout the S-layer with the greatest density being in the C-terminal domain where about 25% of the polypeptide contains one third of the consensus sequences. This domain is rich in Lys and Arg, in contrast to the remainder of the S-layer where these residues are quite sparse. As shown in Figure 2, the majority of the predicted tryptic peptides from Leu_1003_ onwards are smaller than about 40 residues, and are thus well suited to electrospray tandem mass spectrometry (ES-MS/MS) whereas many of those in the N-terminal domain are much larger and are therefore expected to be far less tractable to proteomic analysis. We decided, therefore, to focus our efforts on defining glycosylation in the C-terminal domain by performing nano-LC-MS/MS analyses of in-gel tryptic digests (Figure 3) of the S-layer and manually searching the resulting MS/MS data for potential C-terminal glycopeptides. First, we identified spectra containing fragment ions suggesting the presence of sugars. Then, promising MS/MS data were examined for the presence of peptide sequence ions that could be attributed to predicted tryptic peptides in the C-terminal domain. Finally, glycopeptide structures were deduced taking into account likely peptide and glycan compositions, the latter assignment being assisted by knowledge of the glycan on the cytochrome b_558/566_ of *S. acidocaldarius* (Figure 1). Additionally, all the mass spectra acquired in, and adjacent to, the elution windows of the identified glycopeptides were examined for evidence of molecular ions consistent with glycoforms whose abundance and/or m/z values had precluded their selection for MS/MS analysis by the automatic software. The results of these analyses are presented in the following sections.

### 3.2. Evidence That T-26 and T-24 Are Glycosylated

**Figure 4:**
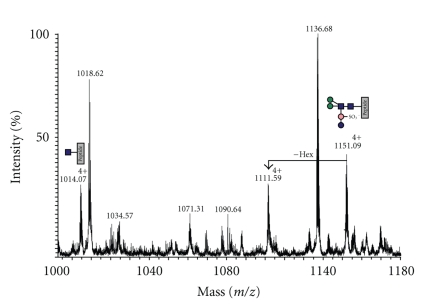
Summed mass spectra recorded between 46 and 52 min from an on-line nanoLC-ES-MS/MS analysis of an in-gel tryptic digest of the S-layer. The quadruply charged signals at* m/z *1014.07, 1111.59 and 1151.09 are molecular ions of glycopeptides of sequence GAGVVEFLLTAYPYTGNITFAPPWFIAENVVK carrying the glycans shown in the annotations. Unassigned signals are molecular ions of peptides from elsewhere in the S-layer.

**Figure 5:**
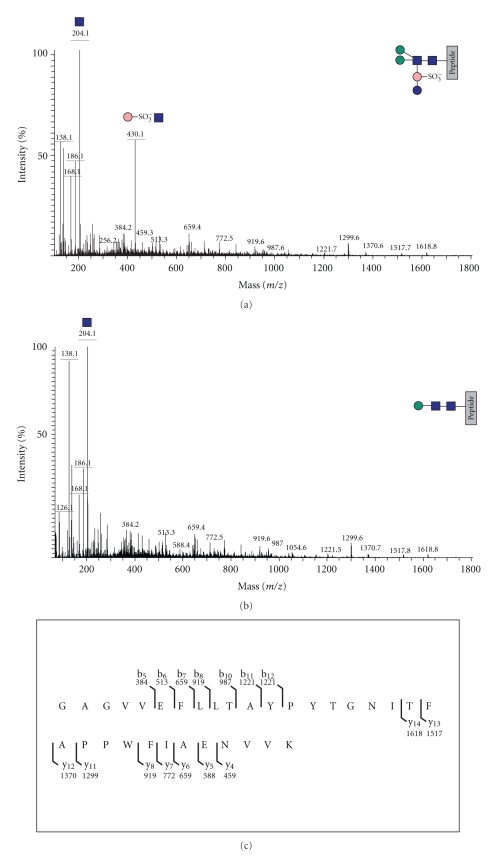
MS/MS of m/z 1151.09 (a) and m/z 1014.07 (b) (see mass spectrum in Figure 4). Note the diagnostic fragment ions for HexNAc in both spectra and for HexNAc-QuiS in (a). The peptide sequence ions are identical in (a) and (b), and their assignments are shown in (c).

Following on-line nano-LC-ES-MS analysis of the S-layer tryptic digest and manual interpretation of the resulting data, a number of multiply charged molecular ions were observed whose product ion spectra were indicative of peptide glycosylation. Once recognised, related glycoforms were identified by summation across the appropriate nano-LC elution time. The summed mass spectrum of components eluting between 46 and 52 min is shown in Figure 4. The signals labelled with their charge states (4+) are glycopeptides corresponding to the tryptic peptide T-26 of sequence GAGVVEFLLTAYPYTGNITFAPPWFIAENVVK (see Figure 2 for designation of tryptic peptides). The [M+4H]^4+^ signals at m/z 1151.09 and 1014.07 were automatically selected for MS/MS, and the resulting spectra are shown in Figure 5. Both are rich in peptide fragment ions that confirm the sequence. Both also are dominated by sugar fragment ions in the low mass region which are diagnostic for HexNAc (m/z 138, 168, 186, and 204) and for HexNAc (204) plus a 226 increment (m/z 430). The latter increment is identical to the mass of the unusual sugar in the *S. acidocaldarius *cytochrome b_558/566_ glycan (Qui-sulfonate, Figure 1). The molecular weights of the quadruply charged signals at m/z 1151.09 and 1014.07 (4600.36 Da and 4052.28 Da) correspond to the molecular weights of the peptide (3483.82 Da) plus a Hex_3_HexNAc_2_QuiS hexasaccharide and a Hex_1_HexNAc_2_ trisaccharide, respectively. The former is consistent with the glycan composition characterised previously for *S. acidocaldarius *cytochrome b_558/566_ (Figure 1). The signal at m/z 1111.59 was not selected for MS/MS but its m/z value is consistent with this component having one fewer hexose than m/z 1151.09 (Figure 4). Without MS/MS data, we are not able to determine whether Glc or Man are absent from the glycan. It is interesting that we do not observe a molecular ion corresponding to Hex_2_HexNAc_2_. 

**Figure 6:**
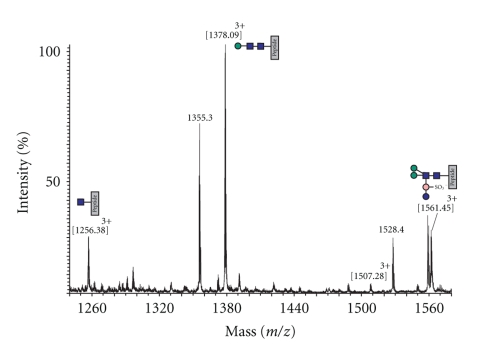
Summed mass spectra of T24 recorded between 48.5 and 50.6 min from an on-line nanoLC-ES-MS/MS analysis of an in-gel tryptic digest of the S-layer. The triply charged signals at m/z 1256.38, 1378.09, 1507.28, and 1561.45 are molecular ions of glycopeptides of sequence LLNLNVQQLNNSILSVTYHDYVTGETLTATTK carrying the glycans shown in the annotations. Unassigned signals are molecular ions of peptides from elsewhere in the S-layer.

We observed similar patterns for all the other tryptic glycopeptides, although there was some variation in relative abundances, particularly of minor components. Some sites had significant amounts of glycans that were truncated to a single GlcNAc. An example is shown in Figure 6, which is the tryptic glycopeptide T-24 having the sequence LLNLNVQQLNNSILSVTYHDYVTGETLTATTK. Triply charged [M+3H]^3+^ molecular ions are observed at m/z 1256.38, 1378.09, 1507.44 and 1561.45 corresponding to glycans of composition HexNAc, Hex_1_HexNAc_2_, Hex_2_HexNAc_2_QuiS, and Hex_3_HexNAc_2_QuiS, respectively. The MS/MS data confirmed the identity of the peptide T-24 (data not shown). For this glycopeptide a relatively abundant ion for the glycopeptides truncated to a single HexNAc was observed (m/z 1256.4). This was not a significant glycoform in the case of T-26.

### 3.3. All Observed Consensus Sequences in the C-Terminal Domain Are Glycosylated

Using the same logic as applied to T-26 and T-24 (see above) we unambiguously identified all the remaining tryptic peptides in the C-terminal domain with the exception of T-29. This is a 66 residue peptide having three consensus sequences (A_1115_SVYY…SSLTK_1180_) and is likely to be too large for the ES-MS experiment. The identified glycopeptides are shown in Table 1 which summarises the m/z values and compositions for glycoforms observed by ES-MS. In an attempt to identify the consensus sites falling within the T-29 tryptic glycopeptide we carried out a chymotryptic digestion. This is not an ideal enzyme for glycoproteomics because it cleaves relatively nonspecifically at large hydrophobic residues as well as at aromatic residues. Hence it yields a very complex digest and consequently the MS and MS/MS data are an enormous challenge for manual interpretation. Fortunately, however, two useful sets of glycopeptide data from the C-terminal domain of the S-layer were revealed by manual inspection. Firstly high quality spectra were found for the C-terminus itself (AGGPVLSEYPAQLIFTNVTLTQ; designated C-1 in Table 1), which includes the consensus sequence at Asn_1390_. MS/MS results (not shown) confirmed the major glycoform was the same as that assigned in the tryptic digest (Table 1) where our evidence had been confined to MS data only. More usefully the chymotryptic digest gave data corresponding to a glycopeptide of sequence TIVPNNTVVQIPSSL which spans the consensus site at Asn_1168_ within T-29 (designated C-2). Once again the glycan profile was similar to other observed sites with the hexa- and trisaccharides being the most abundant components (Table 1).

### 3.4. Sugar Analysis Confirms Man, Glc, and GlcNAc Content

The *S. acidocaldarius *S-layer was analysed for its sugar composition by GC-MS of trimethylsilyl (TMS) methyl glycoside derivatives and the data are shown in Table 2. Consistent with both the MS data described earlier, and the structure reported by Zähringer and colleagues [7], the analysis showed Man, Glc, and GlcNAc as the only observable sugars. No GalNAc was present, confirming the chitobiose core sequence. The Man:Glc ratio is consistent with the mixture of glycans that we found in the glycoproteomics experiments. The unusual quinovose sugar was not observed because sulfonated sugars are not recovered under the experimental conditions used for sugar analysis.

## 4. Discussion

**Figure 7:**
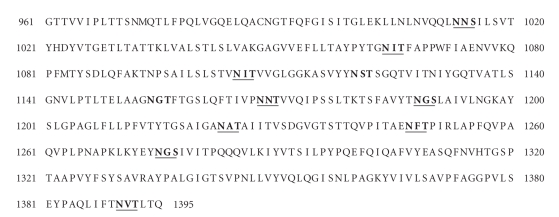
Sequence of the C-terminal domain of the S-layer which was mapped in the glycoproteomics experiments. Consensus sites (in bold) which were shown to be glycosylated are underlined.

In this study we have employed glycoproteomic methodologies to map the C-terminal domain of the *S. acidocaldarius* S-layer, spanning residues L_1004_-Q_1395_. This domain has eleven potential N-linked glycosylation sites, nine of which we have shown to be glycosylated (Figure 7), including Asn_1390_ which is only six residues from the C-terminus. The two sites which were not identified, Asn_1120_ and Asn_1154_, fall within a 66 residue tryptic peptide (T-29) that has three consensus sequences, the third of which (Asn_1168_) was found to be occupied via analysis of a chymotryptic digest of the S-layer. Unfortunately the other two sites were not revealed by this digest. It is noteworthy that we were successful in obtaining good quality data on T-31, which is a 54 residue tryptic glycopeptide carrying two N-glycans (see Figure 7 and Table 1) whose size is not very different from the predicted value for monoglycosylated T-29. We think it is likely, therefore, that our failure to detect signals attributable to the T-29 glycopeptide is probably due to either or both Asn_1120_ and Asn_ 1154_ being glycosylated, in addition to Asn_1168_, thus moving this glycopeptide outside the observable m/z range of the glycoproteomics experiment. Irrespective of whether these two sites are indeed occupied, the glycosylation density in the C-terminal domain is quite remarkable, averaging one glycosylation site for each stretch of 30–40 residues. 

Each of the observed glycosylation sites was found to be heterogeneously glycosylated with a family of glycans, the largest of which has a composition consistent with it having the sequence of the tribranched hexasaccharide found in cytochrome b_558/566_ of *S*.* acidocaldarius* (Figure 1, [7]). The other members of the family appear to be biosynthetic precursors of this glycan. The most abundant is Man_1_GlcNAc_2_ (see annotations on Figures 4 and 6). In addition, a nonextended GlcNAc is a minor component at some sites (Figure 6), and a pentasaccharide lacking one of the hexoses of the mature glycan was also observed (Figures 4 and 6). Interestingly Man_2_GlcNAc_2_ was only observed as a very minor component at a single site (T-34, Table 1), suggesting that either the second mannose is added after the 6-sulfoquinovose, or addition of the latter is rapid compared with the second mannosylation. It is not surprising that the S-layer and the cytochrome share a glycan sequence because conservation of N-glycosylation within a species appears to be characteristic of *Archaea* [24]. Moreover, the presence of a family of biosynthetically related glycans is not unexpected since the S-layer of *H. volcani* exhibits a similar phenomenon [27]. 

With the exception of the two halophiles described in the Introduction, whose glycans are linked via glucose, all known archaeal glycans are attached to Asn via GalNAc or GlcNAc. Interestingly, *S. acidocaldarius* is the only species characterized so far whose glycans are linked via chitobiose (GlcNAc*β*1-4GlcNAc), the core disaccharide shared by the N-linked glycans of *Eukarya*. Moreover the tribranched topology of the *Sulfolobus* glycan is reminiscent of eukaryotic glycans which are usually multiantennary. 

Sulfolobus spp. are developing into model organisms for studies on *Eukarya*-like mechanisms of transcription, translation, and cell division, and the vast amount of recently established genetic tools now make these organisms a prime choice to study these phenomena. The glycan structural information reported in this paper will facilitate the application of genetic tools to the elucidation of the N-glycosylation pathway in *S. acidocaldarius*. It will be very interesting to establish whether the commonalities in core structure between the glycans of *S. acidocaldarius* and those of *Eukarya* are mirrored in the biosynthetic pathways. Moreover, identification of the glycosylation enzymes of *S. acidocaldarius* could lead to interesting biotechnological applications.

## Figures and Tables

**Table 1:** Glycopeptides observed in LC-MS/MS analyses of proteolytic digests of the S-layer from *S. acidocaldarius*. Tryptic glycopeptides are designated T24, T26 and so forth based on the *in silico* digest shown in Figure 2. Two chymotryptic glycopeptides derived from the C-terminal domain of the S-layer are designated C-1 and C-2.

Glycopeptides designation	Peptide Residues	Theoretical Peptide [M+H]^+^	Observed glycopeptide (*m/z*)	Deduced glycopeptide [M+H]^+^	Glycan Modification
T24	1004–1035	3566.00	[1561.5]^3+^	4682.5	Hex_3_HexNAc_2_Qui (SO_3_^−^)
			[1507.5]^3+^	4520.5	Hex_2_HexNAc_2_Qui (SO_3_^−^)
			[1377.7]^3+^	4131.2	Hex_1_HexNAc_2_
			[1256.4]^3+^	3767.2	HexNAc_1_
T26	1048–1079	3484.82	[1151.6]^4+^	4603.4	Hex_3_HexNAc_2_Qui (SO_3_^−^)
			[1111.3]^4+^	4442.2	Hex_2_HexNAc_2_Qui (SO_3_^−^)
			[1014.1]^4+^	4053.0	Hex_1_HexNAc_2_
T28	1093–1114	2141.22	[1087.3]^3+^	3259.9	Hex_3_HexNAc_2_Qui (SO_3_^−^)
			[1033.2]^3+^	3097.7	Hex_2_HexNAc_2_Qui (SO_3_^−^)
			[904.0]^3+^	2710.0	Hex_1_HexNAc_2_
T30	1181–1198	1855.00	[992.2]^3+^	2974.6	Hex_3_HexNAc_2_Qui (SO_3_^−^)
			[938.1]^3+^	2811.0	Hex_2_HexNAc_2_Qui (SO_3_^−^)
			[809.0]^3+^	2425.0	Hex_1_HexNAc_2_
T31	1228–1281	5445.19	[1537.0]^5+^	7681.0	[Hex_3_HexNAc_2_Qui (SO_3_^−^)]_2_
T34	1272–1288	1980.04	[1033.5]^3+^	3098.6	Hex_3_HexNAc_2_Qui (SO_3_^−^)
			[979.5]^3+^	2936.5	Hex_2_HexNAc_2_Qui (SO_3_^−^)
			[904.2]^3+^	2709.5	Hex_2_HexNAc_2_
			[850.1]^3+^	2548.4	Hex_1_HexNAc_2_
T37	1364–1395	3407.85	[1509.7]^3+^	4526.3	Hex_3_HexNAc_2_Qui (SO_3_^−^)
			[1325.7]^3+^	3976.2	Hex_1_HexNAc_2_
C-1	1374–1395	2320.09	[1146.6]^3+^	3437.8	Hex_3_HexNAc_2_Qui (SO_3_^−^)
C-2	1164–1178	1582.71	[1350.6]^2+^	2700.2	Hex_3_HexNAc_2_Qui (SO_3_^−^)
			[1075.6]^2+^	2150.2	Hex_1_HexNAc_2_

**Table 2:** GC-MS analysis of TMS methyl glycoside sugar derivatives obtained from *S. acidocaldarius *S-layer glycoprotein. Note that for experimental reasons GlcNAc recoveries are always poor in sugar analysis experiments. We consider it likely that this is the reason for the GlcNAc:Mannose ratio being much lower than expected from the LC-ES/MS/MS data, although we cannot rule out the possibility that other mannose-containing polymers are present in the sample.

Elution time (min)	Characteristic fragment ions	Assignment	Relative amounts
12.85	204, 217, 305, 361	Man	1
14.62	204, 217, 305, 361	Glc	0.38
18.04	173, 259, 314	GlcNAc	0.22
